# Accuracy evaluation of surface registration algorithm using normal distribution transform in stereotactic body radiotherapy/radiosurgery: A phantom study

**DOI:** 10.1002/acm2.13521

**Published:** 2022-01-05

**Authors:** Haenghwa Lee, Jeong‐Mee Park, Kwang Hyeon Kim, Dong‐Hoon Lee, Moon‐Jun Sohn

**Affiliations:** ^1^ Department of Neurosurgery Neuroscience, & Radiosurgery Hybrid Research Center Inje University Ilsan Paik Hospital, College of Medicine Goyang Republic of Korea; ^2^ Department of Medical Physics Memorial Sloan Kettering Cancer Center New York New York USA

**Keywords:** Normal distribution transform algorithm, optical surface imaging, surface registration algorithm

## Abstract

**Purpose:**

To evaluate a feasibility of normal distribution transform (NDT) algorithm compared with the iterative closest point (ICP) method as a useful surface registration in stereotactic body radiotherapy (SBRT)/stereotactic radiosurgery (SRS).

**Methods:**

Point cloud images using the 3D triangulation technology were obtained from a depth camera‐based optical imaging (OSI) system equipped in a radiosurgery room. Two surface registration algorithms, NDT and ICP, were used to measure and compare the discrepancy values between the reference and the current surfaces during the positioning of the patient. The performance evaluation was investigated by calculating the registration error and root‐mean‐square (RMS) values for the surface model, reposition, and target accuracy, which were analyzed statistically using a paired *t*‐test.

**Results:**

For surface model accuracy, the average of the registration error and RMS values were measured as 3.56 ± 2.20 mm and 6.98 ± 1.89 mm for ICP method, and 1.76 ± 1.32 mm and 3.58 ± 1.30 mm for NDT method (*p* < 0.05). For reposition accuracy, the average registration error and RMS values were calculated as 1.41 ± 0.98 mm and 2.53 ± 1.64 mm using ICP method, and 0.92 ± 0.61 mm and 1.75 ± 0.80 mm using NDT method (*p* = 0.005). The overall target accuracy using the NDT method reduced the average of the reposition error and overall RMS value by 0.71 and 1.32 mm, respectively, compared to the ICP method (*p* = 0.03).

**Conclusions:**

We found that the surface registration algorithm based on NDT method provides more reliable accuracy in the values of surface model, reposition, and target accuracies than the classic ICP method. The NDT method in OSI systems offers reasonable accuracy in SBRT/SRS.

## 1 INTRODUCTION

For the success of fractionated stereotactic body radiation therapy (SBRT)/stereotactic radiosurgery (SRS), an accurate and reproducible patient setup is important.^1^, ^2^ Various devices are used in radiotherapy to improve the accuracy of patient setups. Image‐guided radiotherapy (IGRT) is the most commonly used important technique for achieving patient setting accuracy.^1^, ^3^ IGRT widely include X‐ray imaging, cone‐beam CT, and ultrasound to visualize the internal structure of a patient and to match the patient setup to the initial planned setting.^1^, ^3^ Despite the benefits of IGRT techniques, applications of optical surface imaging (OSI) systems are increasing^4^, ^5^, ^6^, ^7^, ^8^, ^9^, ^10^, ^11^, ^12^ because this system provides accurate patient setup and real‐time monitoring using real‐time 3D surface imaging techniques without an additional dose.^5^ OSI system is used to match the current surface image obtained from the 3D cameras to reference surface and to calculate the couch translations needed to correct a patient's position. The OSI system is also known as surface‐guided radiation therapy (SGRT). Although the high interest in surface imaging, there are only four commercially available OSI systems. The OSI technology, registration algorithm, and performance results used in the four commercial systems are summarized in the following paragraph.

The GALAXY (LAP Co., Germany) system is based on laser camera technology and uses an iterative closest point (ICP) algorithm as the registration algorithm.^7^ In a study using this system, the registration error values for reposition accuracy with a rigid phantom were found to be less than 1 mm in most measurements.^7^ The average root‐mean‐square (RMS) value for reposition accuracy of six different tumors in a patient is approximately 5 mm.^8^ IDENTIFY (Varian Co., USA) utilizes a rigid ICP algorithm with a time‐of‐flight camera technology for accurate patient setup. The registration error for this system has been reported to be less than 1 mm for a translation matrix and less than 1 mm for a rotation matrix.^9^ In surface guidance C‐RAD products (C‐RAD Co., Sweden), there are Sentinel and Catalyst products. The Sentinel is a laser surface scanning system and the catalyst comprises one or three structured light cameras. In evaluated results for reposition accuracy using the Sentinel system, the RMS values are reported within 4 mm.^10^ The Catalyst system has registration error values for reposition within 2 mm.^11^, ^12^ The AlignRT (vision RT Co., UK) combines the surface information from two or three stereo cameras, and then performs the various ICP registration algorithms using phantom.^6^ The registration error values between the phantom positions recorded by the surface imaging system and by an infrared optical tracking system were within 1 mm in translation and 1${}^{\circ }$ in rotation.^13^ Various OSI technologies, such as laser camera, time‐of‐flight camera, structured light, and stereo camera are used to develop the OSI system. ICP method is commonly utilized as the surface registration algorithm in most commercial SBRT/SRS systems.^6^, ^7^, ^8^, ^9^, ^10^, ^13^, ^14^ There are insufficient studies on the application of various surface registration algorithms including NDT method for SBRT/SRS.

The ICP algorithms are widely used in many fields, especially in robotics and computer vision, minimizing the difference between the current point cloud and the reference data.^15^, ^16^ Although the rigid ICP algorithm can easily handle 3D surface data, NDT algorithm takes five to nine times faster than ICP to calculate a suitable solution owing to the process of not using the correspondence information between points obtained from previous iterations.^17^, ^18^ In addition, ICP is sensitive to noise and outliers and requires data storage. The normal distribution transform (NDT) algorithm is an alternative to overcome the limitations of ICP method. The NDT algorithm for mobile robot system was developed based on 2D laser scan registration,^19^ and was later expanded to a 3D registration algorithm^20^ that can be applied to various technologies. The NDT algorithm is a point‐to‐distribution method that performs registration using data scans and normal distributions. Because the NDT algorithm performs calculations without searching for the nearest point or storing raw data from the reference data, it has low computational complexity and can significantly reduce the amount of memory required. In addition, several studies^21^, ^22^ in robotics and computer vision fields have reported that NDT has smaller translation errors and computation time than ICP. Thus, in SBRT/SRS field, it is necessary to investigate the possibility of NDT method in the surface registration algorithm for accurate patient setup.

We investigated the usefulness of the 3D‐NDT algorithm^20^ for the surface registration algorithm in our OSI system by using a surface model, repositioning, and target accuracies. We evaluated the performance of the registration error and RMS values to calculate the three types of accuracy. The ICP algorithm^7^, ^19^ was used as a reference to compare the NDT method.

Ultimately, this study is to evaluate the overall registration errors of two different surface registration algorithms in the applications of patient setup using phantom for the improvement of positioning and targeting accuracies during the treatment, and also to assess whether NDT algorithms, as well as ICP algorithms, were useful surface registration algorithm.

## 2 MATERIALS AND METHODS

### 2.1 Customized optical surface imaging system and phantoms

Our radiosurgery room was originally been used with a dedicated LINAC radiosurgical device (Novalis, BrainLAB, GmbH, Munich, Germany) and an infrared diode tracking system (EacTrac, BrainLAB) for the implementation of skin fiducial marker‐based IG‐SBRT. The supplementary built‐in OSI system was installed with three 3D depth cameras (ASTRA S, ORBBEC, China) mounted on the ceiling of a radiosurgery room for real‐time patient setup accuracy improvement and monitoring. The 3D depth camera includes a pattern projector, an infrared camera, and an RGB camera. Three 3D depth cameras, which are located on the left, right, and center of the ceiling, are connected to a desktop (16 GB RAM, GTC 1080Ti, Window10) that runs the software for point cloud data acquisition. The three 3D cameras were fixed in a straight line with a rod, approximately 70 mm from the ceiling. The distance between the right and left 3D cameras was 2200 mm. The 3D camera has 640 × 480 pixels, and its range is 0.4–2 m. Our OSI system uses a 3D triangulation method^4^, ^23^ to find corresponding points in three 2D images taken from the three 3D cameras and provide a 3D vertex map. The map obtained is described in a PLY format.

Customized human torso phantoms were used for accuracy evaluation. In this phantom, a spine‐shaped phantom and a cube phantom were equipped. A cube phantom was used to calculate the target accuracy. The size of the cube phantom was 450 mm in length, 450 mm in width, and 400 mm in height. The EBT3 Gafchromic films^24^ with a 30 × 30 mm area were placed at the center of the film cube phantom in the vertical or lateral plane direction. The irradiated film was scanned in the scanner (Expression 12000XL, Epson, Japan) and RIT software (Radiological Imaging Technology, USA).^25^ Using the iPLAN planning platform (Brainlab GmbH), a spherical target of 1.5 mm diameter was modeled to a 1.5 Gy dose with 100% equivalent capacity for single arc and cone collimation.

### 2.2 Process and characteristics of surface registration algorithm framework

**FIGURE 1:**
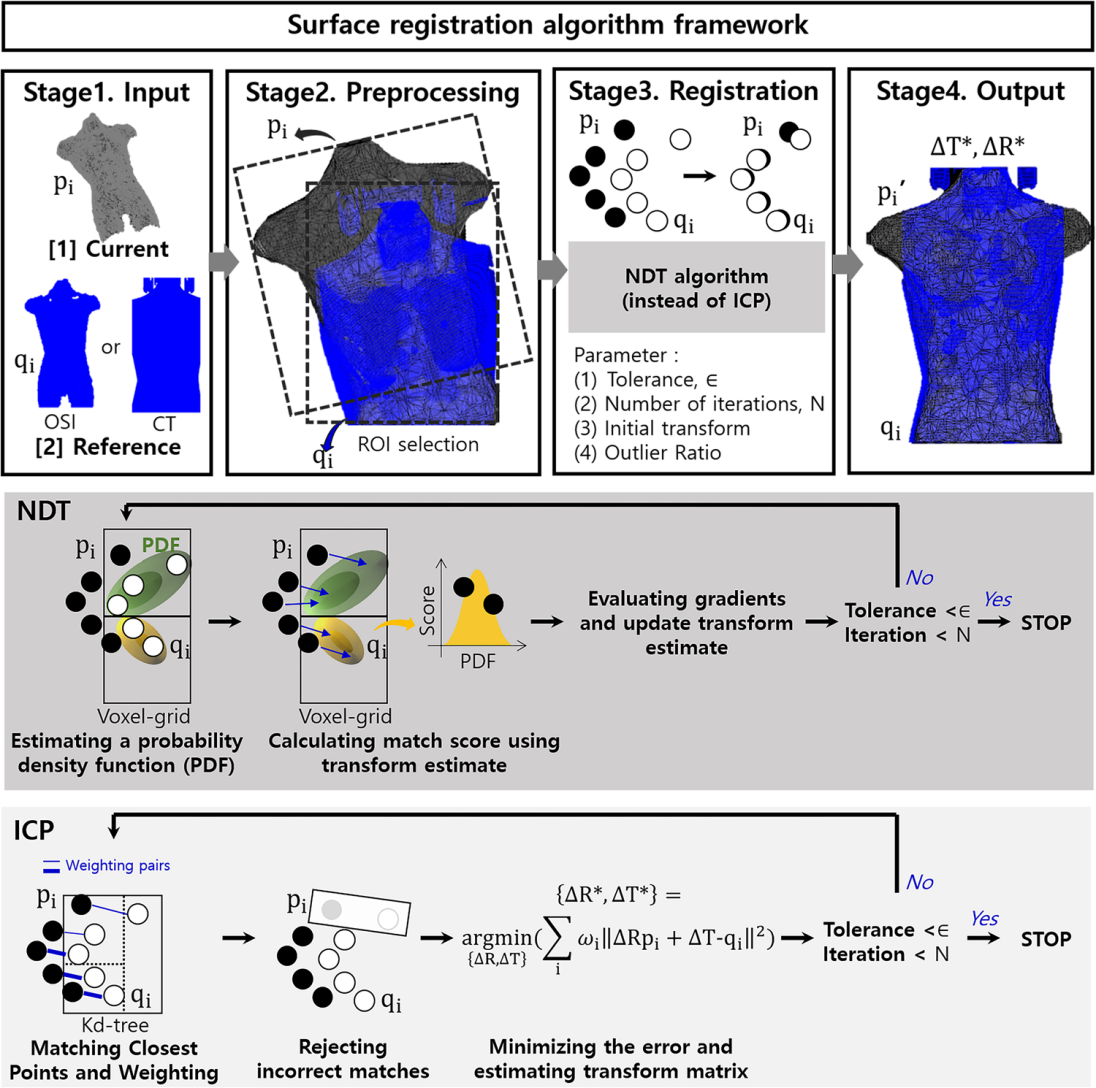
. The overall flowchart of surface registration algorithm using normal distribution transform (NDT) algorithm. The NDT was used in Stage 3, similar to the iterative closest point (ICP) algorithm

The surface registration algorithm is a program that helps patients to be treated at any time in the same position. The surface registration algorithm based on the 3D‐NDT algorithm^20^, ^21^ comprises four stages, as shown in Figure 1. Initially, the current ($p_{i}$) and reference ($q_{i}$) surfaces were input into the proposed algorithm. The current surface represents the point cloud image captured from the current 3D vertices map, and the reference surface was an OSI surface or a CT surface.^4^ The vertex map obtained from our OSI system at the first fraction is called the OSI surface. The CT surface is data obtained by converting a CT DICOM into point cloud data such that it can be calculated using the surface registration algorithm. The following paragraph illustrates this process. Second, we removed outliers from the two surfaces, and then the two preprocessing surfaces were obtained using a user‐defined region of interest (ROI). The user‐defined ROI provides more accurate surface matching by limiting the two preprocessing surfaces to the information contained within the ROI.^9^ The selected ROI included a rectangular surface area of about 600 × 380 × 160 mm^3^ of the respective abdomen. Third, the NDT algorithm is applied to the preprocessed reference and current surfaces. The parameters for this method were selected with maximum values of accuracy that were described in section 4.1. The NDT starts by transforming the reference surface into a normal distribution. We estimate the probability density function for each cell as the normal distribution of inliers, and the uniform distribution of outliers. The match score was then calculated using a transform estimate. Finally, six‐degrees‐of‐freedom (6DOF), which is the transformation difference between the translation (∆T) and rotation matrix (∆R) of the reference surface and the current surface, is obtained as a result. In addition, a transformed surface shifted by a 6DOF value from the current surface was also acquired.

**FIGURE 2:**
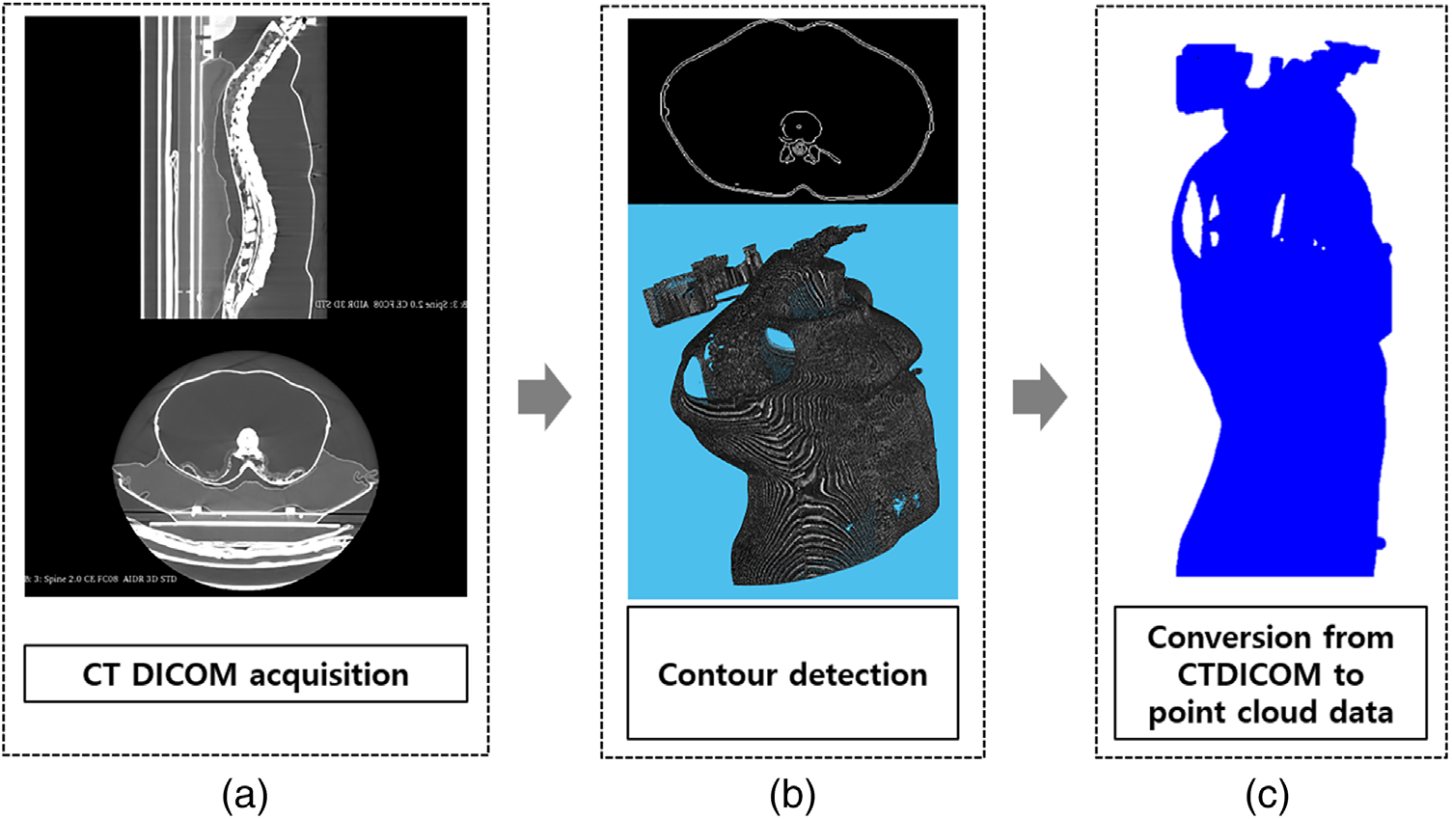
. The conversion process of CT DICOM to 3D point cloud eventually obtained CT surface image

As aforementioned, one of the reference surfaces, the CT surface, was acquired in advance before performing the surface registration algorithm. The process of converting CT images into the CT surface comprised three steps, as shown in Figure 2. First, the CT DICOM images were acquired using a Philips Brilliance Big Bore 16‐slice CT. The image size of the reconstructed plane was 512 × 512 with a pixel pitch of 1.17 mm. In the second step, the contour of the object in the CT data was detected using the segmentation or edge method. In this study, CT data were calculated using the edge method to calculate the voxels, including the contour, as nonzero values in the binary cube. Finally, the position of the voxel with a nonzero value was extracted from the CT 3D image and converted into a PLY format.

### 2.3 Accuracy evaluations for surface registration algorithm

To evaluate the performance of the surface registration algorithm, we measured the registration error and RMS values of the surface model, reposition, and target accuracies. The 6DOF value, which was the result of the surface registration algorithm, was used to calculate the registration errors (or differences) and RMS values. Because the couch could move linearly without rotation, only the physical translation matrix (∆T) among the 6DOF values was used in this study. The physical translation matrix (∆T) is the registration error value, which is the difference between the ground truth transformation (reference, A) and the measured transformation (target, B), and is expressed as follows:

(1) $$\Delta T\;=\left[\begin{array}{c} \Delta t_{LAT}\\ \Delta t_{LNG}\\ \Delta t_{VRT} \end{array}\right]\;=\left[\begin{array}{c} \left|t_{LAT}\left(A\right)-t_{LAT}\left(B\right)\right|\\ \left|t_{LNG}\left(A\right)-t_{LNG}\left(B\right)\right|\\ \left|t_{VRT}\left(A\right)-t_{VRT}\left(B\right)\right| \end{array}\right]\;.$$

Here, $\Delta t_{LAT}$ is distance in the lateral axis between *A* and *B* targets. Also, $\Delta t_{LNG}$ and $\Delta t_{VRT}$ is difference in the longitudinal and vertical between targets. The reasons for using registration errors in the three accuracies are explained in this section. The surface model accuracy can quantify and visualize how the contours of the CT surface (reference) and the current surface (target) match.^4^ Reposition accuracy calculates how different the positions of the OSI surface (reference) and the current surface (target) are. The target accuracy measures the difference between the center of the planned target (reference) and the center of the actual irradiated beam (target). As the registration error value decreases for all the three accuracies, the difference between the position of the target and the reference decreases. The RMS values were used to quantify the registration error values for the *x*, *y*, and *z* axes as a single value:

(2) $$\mathrm{RMS}\;=\sqrt{{\left(\Delta t_{LAT}\right)}^{2}+{\left(\Delta t_{LNG}\right)}^{2}+{\left(\Delta t_{VRT}\right)}^{2}}\;.$$

When the RMS decreases, the accuracy improves. Student's *t*‐test was performed using IBM SPSS Statistics version 22.0 to evaluate the three accuracies of the ICP and NDT registration algorithms.

## 3 RESULTS

The ICP algorithm was used as the reference registration method. For a comparison with the NDT algorithm under the same conditions, the ICP algorithm used surface images obtained by preprocessing the proposed registration algorithm. The surface model, reposition, and target accuracies of the ICP and NDT algorithms were evaluated to investigate the accuracy of the patient setup. In all accuracy evaluations, the torso and cubic phantoms were used as a set to acquire surface images from the CT and OSI systems.

### 3.1 Registration error

For surface model accuracy evaluation between the CT and OSI surfaces, the registration error values were calculated (Table 1). In this study, the experiments were repeated five times per algorithm. The mean registration errors were 3.56 ± 2.20 mm for the ICP algorithm and 1.76 ± 1.32 mm for the NDT algorithm.

**TABLE 1:** Registration error values (mean ± STD) for surface model accuracy

	Registration error values (mm)		
Methods	*x*	*y*	*z*
ICP	1.25 ± 0.42	4.25 ± 0.38	5.18 ± 2.47
NDT	1.45 ± 1.04	1.21 ± 0.24	2.62 ± 1.87

Table 2 lists the registration error values for reposition accuracy with different couch shifts. We used the shift values of the couch as the ground truth. The 12 sets were performed with the couch moved 2, 3, 6, and 10 mm on each axis. A total of 36 current surfaces were obtained by repeating three iterations per set. Thirty‐six current surfaces and one OSI surface were used as input data for the surface registration algorithm. When we used the ICP method, the mean registration errors and its standard deviations were 1.66 ± 1.15, 1.39 ± 0.83, and 0.73 ± 0.88 mm for the *x* (lateral), *y* (longitudinal), and *z* (vertical) axes, respectively. The registration errors and standard deviation values using the NDT method were 1.20 ± 0.97 mm on the *x*‐axis, 1.11 ± 0.45 mm on the *y*‐axis, and 0.88 ± 0.39 mm on the *z*‐axis. In both the ICP and NDT algorithms, the registration errors were the lowest when the movement of the couch was 2 mm. These results show that the registration error increases as the movement of the couch increases.

**TABLE 2:** Registration errors (mean ± STD) with different shifts of couch in the *x*, *y*, and *z* axes for reposition accuracy

	Registration error values (mm)					
Registration methods		2 mm	3 mm	5 mm	10 mm	Total
*x* (Lat.)	ICP	0.67 ± 0.05	1.11 ± 0.13	1.36 ± 0.05	3.51 ± 0.05	1.66 ± 1.15
	NDT	0.65 ± 0.06	1.10 ± 0.09	1.34 ± 0.13	1.35 ± 0.62	1.20 ± 0.97
*y* (Long.)	ICP	0.28 ± 0.08	0.32 ± 0.12	1.85 ± 0.07	2.35 ± 0.30	1.39 ± 0.83
	NDT	0.10 ± 0.01	0.16 ± 0.13	0.64 ± 0.27	2.16 ± 0.10	1.11 ± 0.45
*z* (Vert.)	ICP	0.45 ± 0.08	0.97 ± 0.14	1.55 ± 0.12	2.58± 0.08	0.76 ± 0.88
	NDT	0.30 ± 0.13	0.96 ± 0.12	1.24 ± 0.06	1.04 ± 0.16	0.88 ± 0.39

Experiments were repeated three times per algorithm.

**FIGURE 3:**
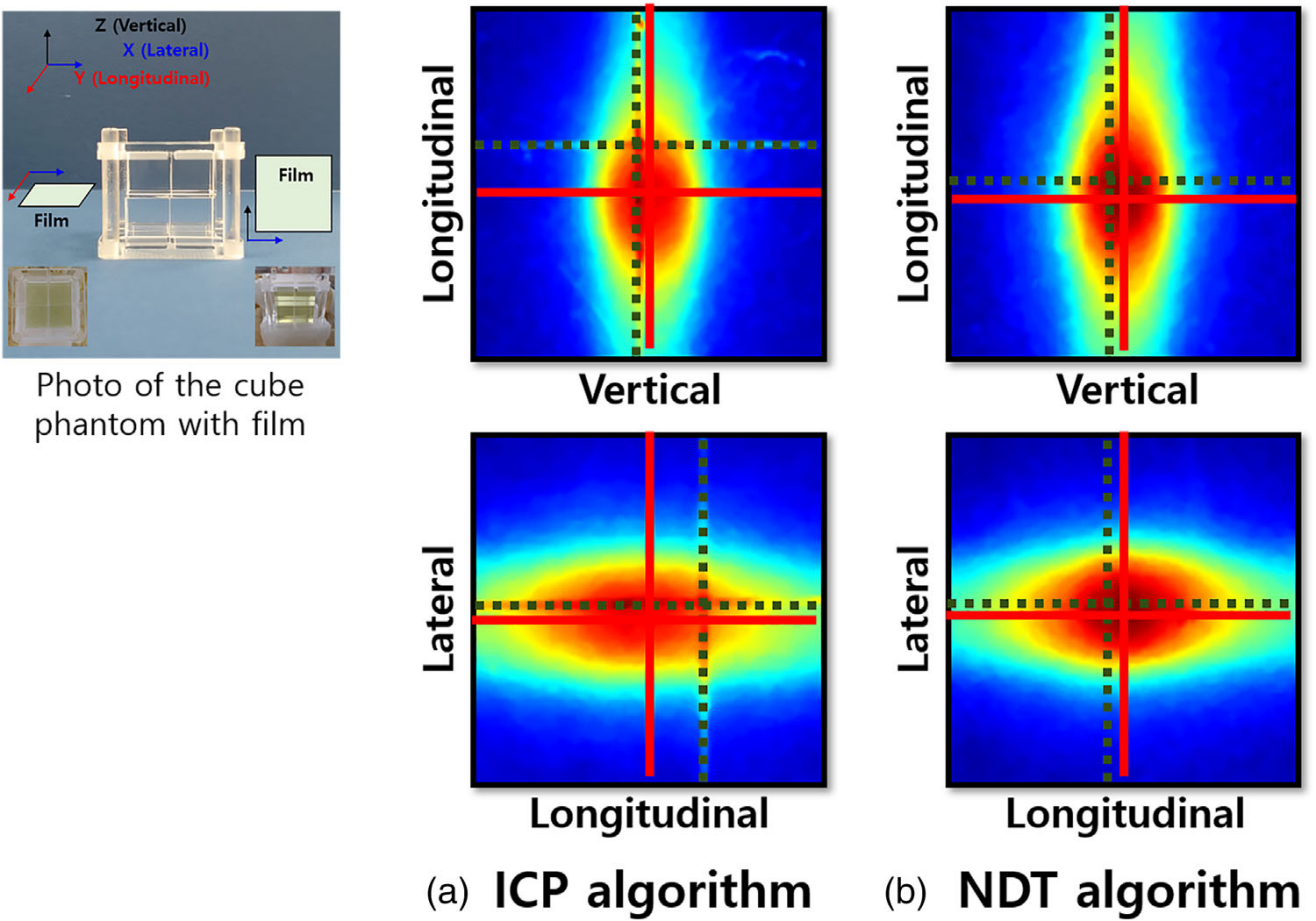
. One case of the film images obtained after repositioning the phantom by (a) ICP and (b) NDT methods. The target accuracy was measured by calculating the distance different between the centers of the cube phantom (dotted line) and real X‐ray beam (solid line)

To measure the target accuracy, the film was mounted vertically or laterally on the cube phantom in the torso phantom. The OSI surface was obtained in the first fraction treatment, and the current surface was obtained by randomly moving the phantom. After the 6DOF values between the current and OSI surfaces were calculated using the ICP or NDT method, the phantom was moved as much as 6DOF to reposition. The beam was irradiated onto a phantom containing the film in the moved position. In one case of the irradiated films, the difference between the center of the planned target (black dotted line) and the center of the X‐ray beam (red dotted line) indicates the target accuracy (Figure 3). The difference values using the ICP method were 0.53 mm on the *x*‐axis, 2.48 mm on the *y*‐axis, and 0.28 mm on the *z*‐axis. When the NDT method was used, the difference was 0.08 mm on the *x*‐axis, 0.32 mm on the *y*‐axis, and 0.22 mm on the *z*‐axis. In these film images, the ICP algorithm exhibits larger displacement differences compared to the NDT algorithm.

Table 3 lists the mean registration error values of the five times obtained for each of the two algorithms. A total of 20 films were irradiated five times in the vertical and lateral directions per algorithm. The mean registration errors for the *x*, *y*, and *z* axes were 1.52 ± 1.18 mm for the ICP algorithm and 0.81 ± 0.92 mm for the NDT algorithm. All registration error results using the NDT algorithm improved better.

**TABLE 3:** Registration error values (mean ± STD) of the irradiated films for target accuracy

	Registration error values (mm)		
Methods	*x*	*y*	*z*
ICP	1.02 ± 1.45	2.34 ± 0.54	1.19 ± 1.09
NDT	0.29 ± 0.20	1.71 ± 1.13	0.43 ± 0.33

### 3.2 RMS value

**FIGURE 4:**
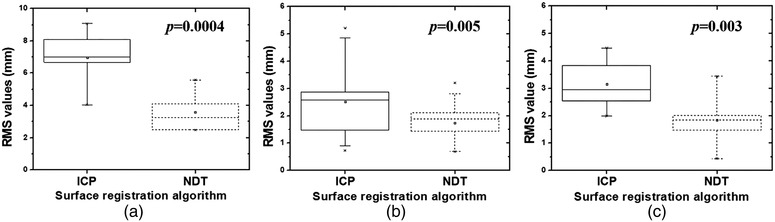
. Boxplots of the root‐mean‐square (RMS) values calculated for (a) surface model, (b) reposition, and (c) target accuracies using the ICP (solid line) and NDT (dotted line) methods. In surface model accuracy, the average RMS values using the ICP and NDT methods were 6.98 ± 1.89 mm and 3.58 ± 1.30 mm, respectively. In reposition accuracy evaluation, the average RMS values were 2.53 ± 1.64 mm for the ICP method and 1.75 ± 0.80 mm for the NDT method. In target accuracy evaluation, the mean RMS value was calculated as 3.16 ± 0.99 mm for the ICP method and 1.84 ± 1.08 mm for the NDT method

The boxplots of the RMS results for the surface model, reposition, and target accuracies were obtained to quantify the registration error values as a single value (Figure 4). The average RMS values of surface model accuracy were 6.98 ± 1.89 mm for the ICP algorithm and 3.58 ± 1.30 mm for the NDT algorithm (Figure 4a). In the *t*‐test of these results for surface model accuracy, the significance value (*p*) was significant at less than 0.05. In the results of reposition accuracy as shown in Figure 4b, the average RMS value was 2.53 ± 1.64 mm for ICP and 1.75 ± 0.80 mm for NDT (*p* = 0.005). The average RMS values calculated using the center difference between the planned target and the irradiated film are 3.16 ± 0.99 mm for ICP and 1.84 ± 1.08 mm for the NDT algorithm (Figure 4c). These results were statistically significant (*p* = 0.03).

## 4 DISCUSSION

We conducted three accuracy experiments to investigate the performance of the proposed NDT method as a surface registration algorithm for our OSI system. This section describes the characteristics of surface registration algorithms, accuracy assessment, and clinical application.

### 4.1 Characteristics of surface registration algorithms

To evaluate the NDT method, the accuracy results, which are commonly chosen as surface registration algorithms for commercialized OSI systems, were compared using the ICP algorithm.^6^, ^7^, ^8^, ^9^, ^10^, ^13^, ^14^ The principle of the conventional ICP algorithm is a point‐to‐point calculation method^26^ that estimates the optimal transformation to overlap two scan data by iteratively minimizing the sum of the distances between the corresponding points. Although the ICP method is easily implemented, this technique is computationally expensive or has low accuracy owing to the nearest point calculation. The NDT algorithm is another popular rigid registration method in computer vision, 3D modeling, and robotic science.^15^, ^27^, ^28^ Because NDT uses a set of distributions transformed from a set of points without searching for the nearest neighbor, there are reports in computer vision and robotics that it has faster computation times and higher accuracy than the ICP method.^20^, ^29^, ^30^, ^31^ However, few studies have used the NDT registration algorithm in the OSI system.

ICP and NDT algorithms have many parameters that can be changed.^20^ To avoid the possibility of various combinations, the following reference combinations were selected. Because the point‐to‐point error metric is the basic metric for ICP method, we selected it as a metric parameter.^32^ For ICP algorithm, a KD‐tree search that can significantly increase the matching speed through the nearest neighbor was used.^6^, ^21^ The NDT method evaluated the speed and accuracy of 10 × 10 × 10, 50 × 50 × 50, and 100 × 100 × 100 to determine the grid size, resulting in that 100 × 100 × 100 mm^3^ was proper. For NDT algorithm, the Newton's method, which is widely used as an optimization method, was chosen.^21^ The zero transform and rotation matrices were used as initial transformations in both ICP and NDT algorithms.^31^ For the two algorithms, there are minimum error and number of iteration as a criterion parameter for stopping iteration. In both the two algorithms, the same minimum errors were set at 0.1 mm and 0.1°.^6^ Registration error values were calculated among 10, 30, 50, 100, and 200 iterations to determine the proper number of iterations, and as a result, most registration error values converged after the number of iterations was 100. So both algorithms decided that the number of iterations was 100.^33^ The average processing time was 2.93 and 0.28 s for the ICP and NDT methods, respectively, in the MATLAB program. Calculations using the NDT method were faster than in the ICP method by setting the size of the grid cell.

### 4.2 Comparison of accuracy evaluation between ICP and NDT algorithms

For performance evaluation, the surface model, reposition, and target accuracies were chosen as the evaluation indexes. The surface model and reposition accuracies have been considered in numerous studies to analyze the performance of the surface registration algorithm for the OSI system.^2^, ^3^, ^4^, ^5^, ^6^, ^7^ We additionally investigated the target accuracy. In all the experimental results of this study, only the translation matrix was evaluated, excluding the rotation matrix, by considering the linear motion of the couch without rotation. The significance of the results for the three accuracies was statistically measured using the Student's *t*‐test.

The surface model accuracy was used to check that the current surfaces derived from the OSI system were in agreement with the original surface from the CT planning images. The CT surface is the clinical standard and is provided to each patient; therefore, we chose it as the reference surface. Converting a CT image to a CT surface involves several steps such as segmenting the surface data and discretizing its values. The mean registration error using the NDT algorithm was 1.76 mm, which was 1.80 mm less than that of the ICP method. The RMS results using the ICP registration algorithm were approximately 4–9 mm, similar to the results of previous studies.^8^, ^10^, ^34^, ^35^ The average RMS value of the NDT algorithm was 3.58 mm, which was approximately 3.40 mm smaller than that of the ICP.

When calculating the reposition accuracy, the movement of the couch was used as the ground truth.^36^, ^37^ These authors found that the mean registration errors for reposition accuracy were 1.41 ± 0.98 mm for ICP and 0.92 ± 0.61 mm for NDT. The mean registration errors using NDT were improved by 0.49 mm. The average RMS values for the ICP and NDT methods were 2.53 ± 1.64 mm and 1.75 ± 0.80 mm, respectively. That is, the average RMS value obtained from the NDT method is also reduced by 0.78 mm compared to that of the ICP method. These results indicate that the NDT algorithm can be set up to the position of the first fraction of the object more accurately than the ICP algorithm. In both algorithms, the registration error and RMS values increased with the increasing movement of the couch. This is because the ICP and NDT algorithms are affected by the initial estimation.^38^

The target accuracy was calculated as the difference between the center of the planned target and the irradiated film. To evaluate the target accuracy, the center of the planned target was calculated as a true value. The average registration error using the NDT algorithm was 0.81 mm, which was 46.71% less than that when using the ICP algorithm. The mean RMS values of the ICP and NDT algorithms were 3.16 ± 0.99 mm and 1.84 ± 1.08 mm, respectively. These results showed that our OSI system using the NDT algorithm effectively improved the film target accuracy. In all three types of accuracy assessments, the significance of the *t*‐test was less than 0.05. All results show that the NDT algorithm can provide an improved surface model, repositioning, and target accuracies.

### 4.3 Clinical feasibility of NDT application

For the SRS/SBRT field, the deviation of the therapeutic couch position indicator was within ±1 mm.^39^ In reference to this, the maximum permissible absolute registration error value for conversion was assumed to be 1 mm. The registration error value using the NDT algorithm was 1.76 mm for surface model accuracy, 0.92 mm for reposition accuracy, and 0.81 mm for target accuracy, and these results are mostly within the acceptable range. The registration error using the NDT method was reduced by 1.80 mm for surface model accuracy, 0.49 mm for reposition accuracy, and 0.71 mm for target accuracy than when using the ICP method. This is because the NDT method can solve the problem by calculating the distribution without information loss from a 3D camera using the normal distribution.

Bert et al. found that the average registration error of surface model and reposition accuracies was 0.50 and 0.95 mm using ICP registration software of the AlignRT system.^4^ Cervino et al. reported that the average registration error of reposition accuracy was 0.93 mm using RANDO head phantom using AlignRT system.^13^ Krell et al. compared the reposition accuracy of various ICP algorithms for phantoms, and reported an average registration error of less than 2 mm.^6^ Our results are of similar level with the previous research's results using the OSI system. Thus, we can conclude that the surface registration for interfractional setup has such inevitable uncertainties compared to the volumetric registration.

While the calculated accuracy in our phantom experiments is precise, the system performance on patients may not reach this precision because rigid transformations might not be appropriate for all sites owing to soft tissue surface deformation. Considering this, future studies will evaluate the NDT algorithm using the patient's surface information.

## 5 CONCLUSIONS

The NDT algorithm was applied as a registration method for accurate patient settings of the OSI system. The results showed that the NDT algorithm can improve the surface model, reposition, and target accuracy compared to the conventional ICP method. Although the algorithm was evaluated only in the environment of rigid deformation without considering the soft tissue surface deformation, we found that the NDT algorithm has the potential to improve the accuracy of OSI systems in SBRT/SRS.

## AUTHORS’ CONTRIBUTION

M‐J. S. agreed to be accountable for all aspects of the work in ensuring that questions related to the accuracy or integrity of any part of the work are appropriately investigated and resolved and approved this version to be published. H. L. conceived conceptualization. H. L. and J‐M. P. carried out the investigation. H. L performed the analysis and writes the manuscript. KH. K. and D‐H. L contributed to revise it critically for important intellectual content. KH. K. contributed to experimental set‐up and dose planning using LINAC and TPS. M‐J. S. and H.L took writing—review and editing.

## CONFLICT OF INTEREST

The authors have no relevant conflict of interest to disclose.
